# Relationship between illness perceptions, coping style and post-concussion symptoms after systemic traumatic injury: A comparison of those with and without mild traumatic brain injury

**DOI:** 10.1177/13591053251395636

**Published:** 2026-01-08

**Authors:** Jacqueline F. I. Anderson

**Affiliations:** 1 The University of Melbourne, VIC, Australia; 2 The Alfred hospital, Melbourne, VIC, Australia

**Keywords:** mild traumatic brain injury, coping, illness perception, post-concussion symptoms, outcome

## Abstract

Research has reported variable findings when investigating the relationship between outcome and coping style and illness perceptions after mild traumatic brain injury (mTBI). The present study investigated 138 individuals who had been admitted to hospital after suffering a traumatic systemic injury, with 91 of these individuals also fulfilling criteria for mTBI and 47 comprising a trauma control (TC) comparison group. Participants were assessed 8 weeks after injury with measures of coping style, illness perception, post-concussion symptoms (PCS) and psychological symptomatology. After controlling for psychological distress and sex, no linear or predictive association was found between any coping style and PCS endorsement for either group. Despite equivalent levels of PCS and general injury-related symptom endorsement (Identity beliefs) between the groups, regression analyses revealed that Identity beliefs significantly improved the prediction of PCS endorsement in the mTBI group, but not in the TC group. The clinical implications of these findings are discussed.

## Introduction

Mild traumatic brain injury (mTBI) is common in civilian and non-professional/-elite athlete populations (Carroll et al., 2014). Despite a longstanding view that full recovery occurs for approximately 80% of individuals by 3 months post-injury (Carroll et al., 2004b), more recently it has been shown over 50% of individuals continue to report incomplete subjective recovery 1 year after injury (Machamer et al., 2022; Nelson et al., 2019). A range of factors have been identified that increase the risk of incomplete longer term subjective recovery, including past psychiatric history (Scheenen et al., 2017a), depression (Iverson, 2005), psychological distress (Anderson and Fitzgerald, 2020; Scheenen et al., 2017a) and female sex (Anderson and Jordan, 2023, 2025; Levin et al., 2021). Coping style and aspects of illness perceptions have also been shown to predict incomplete subjective recovery (Anderson and Fitzgerald, 2020; Snell et al., 2011a).

Coping style and illness perceptions represent pillars of Leventhal’s Common Sense Model (CSM) of health and illness behaviour (Leventhal et al., 1998), which has been used to understand outcome after mTBI (Anderson and Fitzgerald, 2020; Snell et al., 2011a; Whittaker et al., 2007). Coping is a multidimensional concept that can be differentiated into relatively inter-situationally stable ways of handling stressful situations. There two broad coping styles: functional (e.g. seeking help, taking problem-focused action) and dysfunctional (e.g. avoidance, venting; Snell et al., 2011b). Illness perceptions are the ways in which an individual perceives, or personally constructs, their illness with respect to illness identity, timeline, cause, consequences and personal control (Leventhal et al., 1998). The CSM posits that illness perceptions have both a direct and mediating effect on coping styles and outcome. Research examining coping style has found varying support for the notion that an active, problem solving, approach-type coping style is associated with better self-reported outcome (Anderson and Fitzgerald, 2020; Maestas et al., 2014; Scheenen et al., 2017a, 2017b). Most of these studies included individuals with mTBI who had also had previous experience of traumatic injury and/or psychiatric history, which limited the understanding of the role of active coping in managing a specific mTBI event. One study did control for these factors, however and found a significant predictive relationship between active coping and self-reported outcome (Anderson and Fitzgerald, 2020).

Research examining illness perceptions has also shown varying outcomes, with some researchers finding that a belief that an individual will experience negative consequences from the injury to be associated with poor subjective outcome (Hou et al., 2012; Snell et al., 2013; Whittaker et al., 2007). After controlling for the impact of psychological factors, however, others have found no such association (Anderson and Fitzgerald, 2020). Other aspects of illness perceptions have been less consistently associated with subjective outcome. Without controlling for psychological factors, researchers have found that the belief that the injury had an emotional impact on the individual (Snell et al., 2013) as well as a generally negative perception of the injury (Hou et al., 2012) were associated with poor self-reported outcome, but these relationships were not identified after controlling for psychological factors (Anderson and Fitzgerald, 2020). In contrast, it has been shown that the more generic physical symptoms an individual is experiencing that they believe to be directly due to the injury (Identity beliefs) the more likely that the individual will have poorer subjective outcome (Hou et al., 2012; Snell et al., 2013). That is, it is not the absolute number of symptoms the individual experiences that is key, but the number of symptoms they believe to be due to the mTBI that is important. Unlike other reported associations between illness perceptions and outcome, this relationship is present even after controlling for psychological factors (Anderson and Fitzgerald, 2020).

To understand the relationship between subjective outcome and mTBI more fully, it is necessary to consider additional factors that may be affecting this relationship. While psychological factors have been shown to be influential in understanding the linear relationship between subjective outcome and coping style and illness perceptions (Anderson and Fitzgerald, 2020), research suggests that other factors may also be relevant. Firstly, female sex has recently been shown to be significantly associated with poorer subjective outcome after mTBI (Anderson and Jordan, 2023; Levin et al., 2021; Levy et al., 2024). Secondly, general trauma and/or illness factors have been found to impact self-reported outcome. Specifically, individuals who are recovering from a range of conditions that are unrelated to head injury endorse equivalent degrees of symptom severity on the same measures of subjective outcome that are used with mTBI samples (Cassidy et al., 2014; Ettenhofer and Barry, 2012; Laborey et al., 2014; Maruta et al., 2016). It has been suggested that endorsement of poor outcome in non-head injured populations occurs due to the nature of the subjective outcome measures used to measure recovery after mTBI. That is, these post-concussion symptom (PCS) measures comprise a range of physical, affective and cognitive symptoms, the majority of which are generic to a wide range of illnesses and recovering conditions, such as systemic trauma and orthopaedic injury (Anderson, 2021). Consequently, to fully understand the role of a mTBI on subjective outcome after injury, it is important to examine individuals with mTBI and compare them to those who have experienced equivalent injury without a mTBI. No study to date has examined whether individuals with systemic injury and no mTBI differ from those with equivalent systemic injury plus mTBI, with respect to the coping and illness perception factors that predict PCS symptomatology. Nor has any study controlled for sex in examining the relationship of coping and illness perceptions with self-reported outcome after mTBI.

The aim of this study was to investigate the relationship between subjective outcome after mTBI and coping strategies and illness perception factors, while controlling for sex and psychological distress, and contrast these relationships with those of a well-matched control group who have suffered equivalent traumatic injury without TBI. It was hypothesised that active coping style and Identity beliefs would significantly predict PCS reporting in systemically injured individuals with mTBI and systemically injured individuals without mTBI.

## Method

Aspects of the method have been described in previous publications relating to this sample, for example, Anderson and Fitzgerald (2020) and Levy et al. (2024) and are briefly re-presented here.

### Participants

Participants comprised 138 individuals, excluding professional athletes and war veterans, who had suffered any traumatic injury (systemic and/or head) between September 2015 and April 2020, and been consecutively admitted to The Alfred hospital or Royal Melbourne Hospital, Melbourne, Australia, in the preceding 6–12 weeks. Detailed description of the recruitment process and the recruitment decision tree have been reported previously (Anderson and Fitzgerald, 2020; Anderson and Jordan, 2021). All participants were admitted because of bodily traumatic injury – not because they had suffered an mTBI. The mTBI group comprised 91 premorbidly healthy adults (69 male) aged 18–60 years, whose traumatic injury included a head strike, and fulfilled criteria for a mTBI event as defined by the World Health Organisation criteria (Carroll et al., 2004a). This can be briefly summarised as (i) one or more of confusion or disorientation, loss of consciousness for 30 minutes or less; (ii) post-traumatic amnesia less than 24 hours; (iii) Glasgow Coma Scale score of 13–15 after 30 minutes. Excluded individuals were those with: any previous neurological history, (other than two or less previous self-reported concussion events; 81% of the sample had no previous self-reported concussions), sleep disorders, chronic fatigue syndrome, diabetes; any history of heavy alcohol consumption, intravenous or regular Class A drug use; history of any psychotic disorder, bipolar disorder, suicidal ideation, current/recent (during previous 12 months) diagnosis or treatment of depression and/or anxiety and/or post-traumatic stress disorder, no history of treatment with more than 1 concurrent anti-depressant or anxiolytic medication, any previous treatment with lithium; current TBI as a result of physical assault/attack; lack of conversational English fluency. The TC participants comprised 47 premorbidly healthy adults (39 male) aged 18–59 years, whose traumatic injury had not included a head strike and who did not report any symptoms of mTBI; this group had the same exclusion criteria as the mTBI group, with the addition of no previous self-reported concussions. No ethnic group differences existed. All participants provided written informed consent and the project was approved by The Alfred hospital and the Royal Melbourne Hospital Human Research Ethics Committees.

### Measures

*Measure of Post-concussion Syndrome*: The Rivermead Post Concussion Symptoms Questionnaire (RPQ) is a widely used measure of PCS symptomatology. It assesses physical (10 items), psychological (3 items) and cognitive (3 items) symptoms experienced during the past 24 hours (King et al., 1995). Each item is measured on a 5-point Likert scale, with scores of 0 and 1 indicating that the individual believes that symptoms have been no worse since prior to the injury, and scores of 2–4 indicating that symptoms have worsened since prior to the injury. It has been shown repeatedly that responses on the RPQ demonstrate a significant and positive linear correlation with psychological status (e.g. Anderson and Fitzgerald, 2020).

*Measure of coping style*: The Brief COPE (Carver, 1997) was used to measure coping style. The Brief COPE is reliable and can be meaningfully interpreted in terms of three factors in the mTBI population (Snell et al., 2011b). The first factor is a problem-focused/approach factor, the second factor is an avoidant or dysfunctional coping factor and the third factor is a help-seeking or social coping factor.

*Measure of illness perception*: The Illness Perception Questionnaire – Revised (IPQ-R; Moss-Morris et al., 2002) has been shown to be a reliable measure of illness perception in an mTBI population (Snell et al., 2010) and is made up of a number of components. The Identity scale represents the number of miscellaneous physical symptoms the respondent is experiencing that they attribute specifically to the injury; these comprise pain, sore throat, nausea, breathlessness, weight loss, fatigue, stiff joints, sore eyes, wheeziness, headaches, upset stomach, sleep difficulties, dizziness, loss of strength. There are also seven Belief sub-scales, which assess beliefs about: duration of illness (Timeline), extent to which symptoms have a cyclical course (Timeline Cyclical), expected outcomes of the illness (Consequences), extent to which the respondent believes they can personally control the illness/symptoms (Personal Control) or control the illness/symptoms through treatment (Treatment Control), extent to which the respondent understands their illness/symptoms (Illness Coherence) and extent to which the respondent has an emotional reaction to the illness (Emotional Representations).

*Measures of depression, anxiety and post-traumatic stress*: Three widely used, valid and reliable questionnaires of psychological distress were used: The *Inventory of Depressive Symptomatology* (IDS) is a 30-item measure of severity of overall depression (Rush et al., 1996). The *Beck Anxiety Inventory* (BAI) is a 21-item measure of anxiety symptomatology (Beck and Steer, 1993). The *PTSD Checklist for the D*SM*-5* (PCL-5; Weathers et al., 2013) is a 20-item measure of the symptoms of PTSD defined by DSM-5. To facilitate data reduction, a single variable of Psychological Distress was created from standardised performances on the IDS, BAI and PCL-5. The IDS and BAI are 4-point scales (range 0–3), whereas the PCL-5 is a 5-point scale (0–4). Consequently, each response on the PCL-5 was multiplied by 0.75, resulting in all measures having item responses on a 4-point scale (range 0–3). Performance on these measures were then summed together, resulting in a single index of psychological distress, with a possible range of 0–207.

### Procedure

Participants were initially recruited into the study on the ward within 1–4 days after injury, during their inpatient stay. Written informed consent to participate was given following fulfilment of inclusion/exclusion criteria. Following discharge, participants were contacted by phone and attended The Alfred hospital to undertake individual assessment 6–12 weeks after injury. All participants completed the measures in the following order: RPQ, IDS, BAI, PCL, Brief COPE and IPQ-R.

### Data Analysis

Statistical assumptions were investigated prior to data analysis (Tabachnick and Fidell, 2013). Only violations of assumptions will be reported. The RPQ was positively skewed, so non-parametric analyses were undertaken when this variable was included. The False Discovery Rate was used to correct for multiple comparisons. Chi-square analyses and multivariate analyses of variance (MANOVA) were used to compare the groups on background variables. Multivariate analysis of covariance (MANCOVA) was used to compare the groups on coping, illness perception and post-concussion symptoms, while controlling for sex, psychological distress and litigation status. Despite the groups being well-matched on proportion of individuals who were litigants, Spearman’s rank correlation revealed a significant relationship between litigation status and increased likelihood of higher RPQ symptom endorsement for the mTBI group (ρ = 0.258, *p* = 0.014), but no significant association was evident for the TC group (ρ = 0.085, *p* = 0.576). After controlling for multiple comparisons with the False Discovery Rate, litigation status did not significantly correlate with any other variable for either group. Consequently, litigation status was included as a co-variate in all analyses involving the RPQ for the mTBI group. Spearman’s partial correlations were used to examine the linear relationship between the RPQ and coping and illness perception variables while controlling for sex and psychological distress for each group; litigation status was an additional covariate for the mTBI group. Bootstrapped hierarchical linear regression analyses were used to investigate whether illness perceptions independently predicted post-concussion symptom reporting, as this is a recommended robust method to compensate for lack of normality (Field, 2013). In each regression model sex and psychological distress were entered in Step 1, with litigation status also entered at this step for analyses examining the mTBI group; illness perception variables were entered at Step 2. Due to the limited sample size, only those IPQ-R and COPE variables that demonstrated significant partial correlations with the respective RPQ variables were included in these analyses.

## Results

Recruitment pathways for this study have previously been provided in detail (Anderson and Fitzgerald, 2020; Anderson and Jordan, 2021). Details of demographic and injury details are presented in Tables 1 and 2.

**Table 1.** Background demographic details.

Background variable	mTBI (*n* = 91)	TC (*n* = 47)	*p*-Value
Age (years) Mean (sd) Range	37.29 (13.81) 18–60	36.21 (12.67) 18–59	0.650
Sex Female; Male	24% F; 76% M	17% F; 83% M	0.334
Educ. (years) Mean (sd) Range	13.23 (2.30) 10–19	13.28 (2.80) 9–21	0.963
Psych. Distr. Index Mean (sd) Range	28.87 (22.72) 0.80–97.40	24.72 (18.57) 4.40-84.60	0.269
PPIQ Mean (sd) Range	105.84 (9.25) 81–124	104.94 (9.70) 84–125	0.554
Occupation (%) Professionals Managers Trade/clerical Unskilled Students	22.00 16.50 31.90 17.60 12.10	20.00 15.60 35.60 15.50 13.30	0.932
Involved in litigation (%)	11.00	10.60	0.950
Psych history (%) None No dx, med Health Prof, no med Dep/anx	74.70 5.50 13.20 6.60	76.60 2.10 19.10 2.10	0.555

Educ.: education; Psy. Distr.: Psychological Distress Index; PPIQ: Predicted Premorbid IQ; No dx, med: no formal diagnosis, has not seen mental health professional but received medication > 12 months previously; Health Prof, no med: no formal diagnosis, has seen mental health professional, but received no medication > 12 months previously; Dep/anx: previous formal diagnosis of depression/anxiety > 12 months previously.

**Table 2.** Injury descriptors.

Injury descriptor	mTBI (*n* = 91)	TC (*n* = 47)	*p*-Value
Days since injury Mean (sd) Range	61.43 (10.50) 37–85	59.38 (13.18) 41–87	0.251
Injury cause (%) MVA MBA Cycling Fall Sport Other	16.50 15.40 27.50 19.80 6.60 14.30	23.40 17.00 19.10 17.00 10.60 12.80	0.773
GCS Mean (sd)	14.59 (0.63)	-	
LOC (% < 5 minutes)	86.80	-	
PTA (% < 60 minutes)	73.60	-	

MVA: motor vehicle accident; MBA: motorbike accident; GCS: Glasgow Coma Scale score; LOC: loss of consciousness; PTA: post traumatic amnesia.

The groups were well matched on all demographic and injury variables; the proportion of litigants was 11% or less in each group.

Multivariate analysis of covariance (MANCOVA) revealed a main effect of group [*F*(11,115) = 2.190, *p* = 0.019] and sex [*F*(11,115) = 1.995, *p* = 0.035] when comparing performance on the measures of coping and illness perception. There were no significant interactions between group and litigation status or sex. Univariate analyses revealed a number of between group differences. The results of these analyses are presented in Table 3.

**Table 3.** Group comparisons of coping, illness perception and post-concussion symptom data, controlling for sex, psychological distress and litigation status.

Variables	mTBI (*n* = 88) $\bar{X}$ (sd)	TC (*n* = 45) $\bar{X}$ (sd)	*F*-statistic	*p*-Value	Effect size Partial η^2^
Brief cope					
Approach Index	29.37 (6.88)	32.23 (7.82)	3.116	0.080	0.024
Avoidant Index	10.89 (2.74)	11.74 (3.73)	0.973	0.326	0.008
Help Seek Index	8.87 (3.09)	9.60 (3.10)	0.302	0.583	0.002
IPQ-R					
Identity	4.05 (3.79)	5.70 (3.26)	5.519	0.020*	0.042
Timeline	11.04 (4.33)	15.64 (5.47)	12.502	**<0.001**	0.091
Consequences	12.48 (5.29)	16.21 (5.11)	12.105	**<0.001**	0.088
Personal control	21.07 (4.73)	23.09 (4.29)	1.398	0.239	0.011
Treatment control	18.46 (3.74)	19.79 (3.01)	0.051	0.822	<0.001
Illness coherence	21.26 (3.64)	22.19 (3.79)	0.091	0.763	0.001
Timeline cyclical	7.51 (3.39)	8.34 (4.00)	4.403	0.038*	0.034
Emotional representation	11.37 (4.11)	13.66 (5.05)	4.842	0.030*	0.037
RPQ	Median (IQR)	Median (IQR)	U-statistic		r_rb_
Total	9.00 (16.00)	7.50 (17.00)	1896.50	0.369	0.09

r_rb_: rank biserial correlation; bolded values are statistically significant.

* Significant in initial analyses, but not significant after False Discovery Rate correction for multiple comparisons applied.

After controlling for multiple comparisons, the mTBI group demonstrated significantly reduced endorsement on the Timeline and Consequences variables of the IPQ-R relative to the TC group. This indicates that the mTBI sample considered their illness/symptoms would persist for significantly less time and have a significantly smaller impact on their lives than the TC group. Both of these group differences were associated with a medium effect size. There were no significant differences in coping style or post-concussion symptoms between the groups after controlling for multiple comparisons and these analyses were associated with small effect sizes.

Spearman correlations confirmed that both groups demonstrated a significant positive correlation between post-concussion symptoms and psychological distress (mTBI: *p* < 0.001; TC: *p* < 0.001). The mTBI group also showed a significant correlation between post-concussion symptom endorsement and sex (*p* = 0.001), with females more likely to endorse higher levels of post-concussion symptoms; the relationship between these variables for the TC group was trending towards significance (*p* = 0.061). Partial Spearman correlations between post-concussion symptoms and the coping and illness perception variables, while controlling for psychological distress and sex (and litigation for the mTBI group), are presented for each group in Table 4.

**Table 4.** Partial correlations between RPQ Total and IPQ-R and Brief-COPE factors while controlling for psychological distress, sex and litigation status.^a^

Variables	RPQ Total *r*-value		Variables	RPQ Total *r*-value	
	mTBI (*n* = 88)	TC (*n* = 45)		mTBI (*n* = 88)	TC (*n* = 45)
Brief Cope Approach Index Avoidant Index Help Seek Index			IPQ-R		
	0.004	−0.206	Consequences	0.113	−0.043
	0.047	0.092	Personal control	0.189	0.020
	0.145	−0.109	Treatment control	0.130	−0.214
IPQ-R Identity Timeline			Illness coherence	−0.057	−0.238
	0.406***	0.106	Timeline cyclical	0.175	0.177
	0.138	0.013	Emotional representation	0.052	0.185

IPQ-R: Illness Perception Questionnaire – Revised; RPQ: Rivermead Post Concussion Symptoms Questionnaire; bolded values are statistically significant.

a Litigation status is only included as a co-variate for the mTBI group.

*** *p* < 0.001.

There were no significant linear relationships between post-concussion symptoms and either coping or illness perception factors for the TC group. In contrast, despite equivalent levels of PCS in both groups, for the mTBI group there was a significant linear relationship between the number of generic physical symptoms an individual believed were related to their injury (Identity beliefs) and the extent of post-concussion symptom endorsement (RPQ). That is, those individuals who more consistently blamed a wide range of physical symptoms (e.g. sore throat, stiff joints, nausea) they were experiencing on having suffered the mTBI event were more likely to experience a larger number of, or more severe, post-concussion symptoms.

Due to the nature of the RPQ’s scoring system, equivalently elevated scores on the RPQ can occur due to either (i) a larger number of symptoms being endorsed with lower levels of severity per symptom, or (ii) a smaller number of symptoms being endorsed with higher levels of severity per symptom. It is possible, therefore, that the difference between the mTBI and TC groups with respect to the linear relationship between RPQ Total and Identity, despite the groups having equivalent overall scores on the RPQ and Identity measures, could be because the groups demonstrated different response profiles on the RPQ measure. That is, one group may have endorsed more severe symptom expression on fewer items and the other group may have endorsed less severe symptoms on more items, resulting in equivalent scores, but different response profiles. If this occurred, this could underlie the difference in relationship to number of illness symptoms that were endorsed. To investigate this, a between group Mann-Whitney *U* test was conducted to investigate whether the groups differed with respect to the number of items that they endorsed as ‘symptom is more of a problem relative to pre-injury’ (vs ‘symptom is unchanged relative to pre-injury’) on the RPQ. Analyses revealed that the groups endorsed equivalent numbers of items on the RPQ for which they believed that their symptoms were more of a problem following injury [*U* = 1987.50, *n*_1_ = 91, *n*_2_ = 47, *p* = 0.491]. This indicates that the groups had equivalent response profiles on the RPQ as well as equivalent absolute scores on this measure.

In light of the mTBI group’s significant correlation between post-concussion symptom endorsement and number of symptoms related to the injury, bootstrapped hierarchical regression analyses were conducted to determine whether RPQ Total could be independently predicted by Identity for each group. Psychological distress, sex and litigation (for the mTBI group) were included as co-variates due to their known relationship with RPQ performance. The resultant model was significant for the mTBI group [F(4,84) = 27.197, *p* < 0.001] as well as the TC group [F(3,41) = 11.643, *p* < 0.001]. The parameters of these models are presented in Table 5.

**Table 5.** Regression model parameters by group.

Group	Regression analysis	Independent variable	*b* (Confidence intervals)		SE B	*p*-Value
mTBI	Step 1	Constant	−7.925	(−15.707, −0.153)	3.892	**0.046**
		Sex	5.656	(0.441, 10.577)	2.703	**0.044**
		Psych distress	0.236	(0.110, 0.385)	0.063	<**0.001**
		Litigation	5.287	(0.651, 10.050)	2.493	**0.031**
	Step 2	Constant	−8.995	(−15.892, −1.449)	3.610	**0.013**
		Sex	4.500	(−0.024, 8.915)	2.311	0.057
		Psych distress	0.167	(0.060, 0.304)	0.061	**0.008**
		Litigation	5.331	(1.014, 9.530)	2.339	**0.021**
		Identity	1.089	(0.582, 1.554)	0.263	<**0.001**
TC	Step 1	Constant	1.000	(−7.780, 9.746)	4.241	0.811
		Sex	0.784	(−6.595, 6.927)	3.689	0.823
		Psych distress	0.326	(0.195, 0.469)	0.069	<**0.001**
	Step 2	Constant	−0.542	(−8.504, 8.906)	4.128	0.897
		Sex	−1.208	(−9.410, 5.441)	3.744	0.723
		Psych distress	0.331	(0.197, 0.461)	0.068	<**0.001**
		Identity	0.664	(−0.024, 1.137)	0.326	**0.044**

mTBI - Step 1: Adj *R*^2^ = 0.416, Δ*R*^2^ = 0.436 (*p* < 0.001); Step 2: Adj *R*^2^ = 0.544, Δ*R*^2^ = 0.129 (*p* < 0.001); TC - Step 1: Adj *R*^2^ = 0.386, Δ*R*^2^ = 0.413 (*p* < 0.001); Step 2: Adj *R*^2^ = 0.421, Δ*R*^2^ = 0.047 (*p* = 0.067); Sex: M coded as 1, F coded as 2; PSQI: Pittsburgh Sleep Quality Index; MFI: Multidimensional Fatigue Inventory; bolded values are statistically significant.

While identity was a significant predictor of RPQ endorsement for both groups, the addition of Identity to each regression model only significantly improved the resultant regression model for the mTBI group (*p* < 0.001), indicating that the addition of Identity over and above sex and psychological distress did not significantly improve the goodness of fit of the regression model for the TC group. For both groups psychological distress significantly predicted RPQ performance. In contrast, sex did not predict RPQ performance for the TC group, but was a significant predictor in Step 1 of the mTBI model and approaching significance in the final regression model for this group. The final model predicted 54% of the variance in RPQ performance for the mTBI group (Adj *R*^2^ = 0.544) and 42% of the variance for the TC group (Adj *R*^2^ = 0.421). Despite a lack of significant relationship between the Approach Index and RPQ performance, due to the apriori hypothesis that Approach coping would significantly predict RPQ endorsement for both groups, a bootstrapped hierarchical regression analysis was conducted for these variables, with psychological distress, sex and litigation status (mTBI group only) included as co-variates. The resultant models were significant for both the mTBI [*F*(4,84) = 16.228, *p* < 0.001] and TC groups [*F*(3,41) = 10.600, *p* < 0.001], but Approach coping did not significantly contribute to either model, nor improve the model fit for either the mTBI (Approach: *p* = 0.858; Δ*R*^2^ = 0.000, *p* = 0.884), or TC (Approach: *p* = 0.229; Δ*R*^2^ = 0.023, *p* = 0.200) group.

## Discussion

This study contrasted and compared the performance of a mTBI and well-matched TC group to better understand the relationship between coping style, illness perceptions and post-concussion symptom expression approximately 8 weeks after mTBI. In contrast to expectations, after controlling for psychological distress and sex, approach coping style was not significantly associated with PCS reporting in the mTBI or TC group, nor did it independently predict PCS performance for either group. Similarly, but consistent with expectations, neither avoidant coping nor help-seeking coping demonstrated a significant linear association with PCS endorsement for either group. In contrast and as predicted, the mTBI group demonstrated that the more symptoms that an individual experienced, which they attributed to their injury (Identity beliefs), the more likely they were to report PCS symptoms (RPQ) approximately 8 weeks after injury. Despite both groups demonstrating equivalent levels of endorsement on both the Identity and RPQ measures, however, the TC group showed no equivalent linear relationship between these variables. That is, endorsement of injury-related symptoms was unrelated to endorsement of PCS symptoms in the TC group after controlling for multiple comparisons. Further, whereas the inclusion of Identity beliefs, in addition to sex, psychological distress and litigation status, significantly improved the ability of the regression model to predict PCS reporting in the mTBI group, it failed to significantly improve model fit in the TC group. Thus, while Identity significantly independently predicted PCS performance in each group, it was only in the mTBI group, that this measure improved the prediction of PCS reporting above the predictive contribution of the co-variates.

The lack of predictive relationship between approach coping style and PCS endorsement in the mTBI group, after controlling for psychological distress, sex and litigation suggests that sex is an important component of this predictive model. Previous research has demonstrated that approach coping style is predictive of PCS endorsement, while controlling for psychological distress and litigation (Anderson and Jordan, 2023), but the current findings indicate that this predictive relationship disappears when sex is added as a covariate. Thus, it appears that sex is an influential factor in the relationship between approach coping and PCS reporting, potentially related to its linear association with the RPQ (mTBI: *r*_pb_ = 0.386, *p* < 0.001; TC: *r*_pb_ = 0.309, *p* = 0.037), as sex did not demonstrate a bivariate linear relationship with Approach coping (mTBI: *r*_pb_ = −0.057, *p* = 0.591; TC: *r*_pb_ = 0.184, *p* = 0.216). From a clinical perspective, this indicates that higher levels of approach coping style do not independently predict lower levels of PCS reporting as has previously been described. Rather, both the sex of the individual and their psychological status are important factors and may mediate this relationship. While the lack of direct relationship between Approach coping and PCS is unexpected in the context of Leventhal’s model of health and illness behaviour, it does not mean that the CSM is irrelevant in the context of mTBI. Rather, other variables, such as sex may be particularly influential in mediating this relationship in the mTBI population. Due to the lack of participants, structural equation modelling was not possible in the current study, but future work investigating the model’s predicted relationship between illness perceptions and coping style would continue to be useful. Elaboration of these relationships would improve our understanding of the way in which these variables relate to one another, other variables such as sex and psychological distress, and outcome. The clinical implications of understanding these mechanistic relationships better would likely be substantial, as has occurred for other conditions (e.g. Knowles et al., 2014).

The finding that Identity beliefs independently predicted level of PCS endorsement, over and above psychological distress and sex, for both groups was consistent with expectations and with the CSM. As the associated regression model fit only improved with the addition of Identity for the mTBI group, however, this suggests that the relationship between Identity and PCS reporting may not be equivalent between the two groups. At a clinical level, this means that for the mTBI group, knowing that an individual believes that more of their physical symptoms are due to their injury will make it more likely that they will endorse greater amounts of PCS, over and above the contribution of their psychological status and sex. Given that individuals with higher levels of PCS at 3 months after injury are also likely to have poor longer-term outcome (Fordal et al., 2022), these results suggest that individuals who blame more of their general symptoms on the mTBI may warrant consideration for earlier intervention, particularly if they are female and are experiencing psychological distress. In contrast, for the TC group, having this additional information will not make predicting whether they will have higher levels of PCS endorsement more accurate. A clinician will be equivalently accurate by considering only the individual’s psychological status and sex.

In the post-acute period, PCS endorsement is no longer associated with more objective measures of underlying neurological recovery (Anderson, 2021). It has been suggested that, aside from the subjective cognition items (Levy et al., 2023), PCS endorsement is more indicative of general malaise than mTBI-specific complaint by 8 weeks post-injury (Anderson, 2021). Sixty-five per cent of Identity belief symptoms are also generic symptoms that are not part of the PCS constellation (i.e. pain, sore throat, breathlessness, weight loss, stiff joints, sore eyes, wheeziness, upset stomach, loss of strength). Consequently, the current study’s finding that the quantity of general physical symptoms an individual has that they believe are due to their injury independently improves the prediction of whether they are reporting general malaise symptoms (PCS endorsement), for the mTBI group but not the TC group, may have mechanistic implications. Analyses of the pattern of symptom endorsement demonstrated that the group difference was not due to one group endorsing fewer PCS items but with higher intensity than the other; both groups endorsed that their symptoms had worsened post-injury for an equivalent number of symptoms. It is possible, however, that the group difference in regression model parameters is due to the nature of symptom expression in the acute period after injury.

For those who have experienced the wide array of PCS that commonly occur in the acute phase due to mTBI-related neuropathology, their post-acute experience of a wide array of symptoms may be more likely to be experienced as symptoms that are consistent in nature with their acute mTBI-related experiences. In turn this could suggest to the individual that they are not recovering well from their mTBI. In contrast, for individuals without injury-related neurological involvement (TC group) they may be less likely to associate their array of post-acute symptoms with a set of post-concussion symptoms, as PCS were not initially part of the acute injury experience. This notion of chronic symptom experience that is consistent in nature with the original injury type, leading to a view that neuropathological recovery is not occurring, has previously been speculated as a potential mechanism leading to perpetuation of PCS expression after mTBI (Lagarde et al., 2014; Scheenen et al., 2017a). The current findings are consistent with this explanation and therefore have implications for intervention. Specifically, psychoeducation about the change in aetiology of PCS from the acute to post-acute period may support improved outcome by assisting patients to re-frame their post-acute experience and try different coping and adjustment techniques to improve or manage their symptoms.

A further unexpected finding of the present study is that despite equivalent levels of overall PCS symptomatology, the mTBI group believed that their injury would have significantly less consequence for their life and last for a significantly shorter time period than the TC group. Given that both groups were admitted to hospital due to traumatic systemic injury, with diagnosis of mTBI an incidental factor following their admission, it is likely that the groups had similar levels of systemic injury. Consequently, it is puzzling why the mTBI group, which has both systemic and brain injury would believe that their injury would be less likely to impact them and have a shorter duration than the TC group, which is experiencing only systemic injury. One possible reason for this difference may relate to the different messaging associated with recovery from mTBI versus systemic injury. Specifically, it is not uncommon for clinicians to advise their patients that full recovery after mTBI can be expected to occur within days (Rose et al., 2015), despite this not corresponding with the personal experience of life re-engagement of a significant proportion of individuals (Chu et al., 2017). In contrast, systemic injury is typically presented as being associated with a longer period of recovery (months rather than days) as is appropriate for musculoskeletal, internal organ and/or vascular damage (Glancy et al., 1992). Irrespective of the cause, given the medium to large effect size of these group differences, investigation of whether these beliefs in the mTBI group affect recovery is warranted. In particular, if individuals with mTBI have a more optimistic expectation of recovery than individuals with systemic injury and no mTBI, this could result in the groups having differential styles of symptom management and psychological coping during the recovery period. Problematically, this has the potential to be deleterious to outcome for individuals with mTBI, if optimistic recovery expectations are not fulfilled. While entirely speculative on the basis of the current study, such a trajectory provides a possible mechanism to explain chronic PCS expression despite underlying neuropathological recovery and therefore warrants further investigation.

The primary limitation of the current study is the modest sample size. The reduced power of the analyses due to the limited sample size means that real group differences may have been obscured or inappropriately ignored following correction for multiple comparisons. While a larger sample size would address this issue, the current sample size does not undermine the reliability or generalisability of this study’s significant findings. It could be argued that a potential second limitation is that the current pre-morbidly healthy mTBI sample is not representative of the broader mTBI population, which typically has a history of psychiatric disturbance, significant drug and alcohol consumption as well as previous TBIs (Dams-O’Connor et al., 2013). This lack of representation does not undermine the value of the current study, however, as all of these factors are associated with increased risk of poorer outcome after mTBI (Cassidy et al., 2014). Consequently, to understand the specific contribution of mTBI and the associated recovery experience on outcome, it is important to be able to examine this relationship without other known influential factors compromising the data and affecting the inferential conclusions that are drawn. Thus, examining a pre-morbidly healthy group provides an opportunity to understand mTBI-specific consequences of the injury and recovery process, which can then be used to inform our understanding of the broader mTBI population. Finally, the greater numbers of males versus females in each group could also be argued to be a limitation of the study, particularly as sex was included as a co-variate in some analyses. Given that bootstrapping analyses, which were carried out for all statistical investigations that controlled for sex, are considered adequate to deal with the small numbers of females in the present study (Chernick, 2008), it is unlikely that the current findings were significantly compromised, however. Indeed, the proportion of females versus males in the current study reflects mTBI population demographics and could be argued to contribute to the validity of the current sample.

In summary, the present study has found that the previously reported predictive relationship between active coping style and PCS endorsement in individuals with mTBI is absent when sex is included as a co-variate, indicating that active coping style does not independently predict PCS reporting 8 weeks after mTBI. In addition, while the number of symptoms an individual experiences that they believe are related to their injury (Identity beliefs) was independently associated with PCS endorsement in the current mTBI sample, no such association was evident in the TC group. Further, Identity beliefs significantly improved a predictive model of PCS reporting in addition to psychological distress and sex in the mTBI group, but not in the TC group. This differential pattern of association between the groups indicates that although individuals with mTBI have equivalent levels of Identity beliefs and PCS endorsement as TC individuals, the relationship between these variables differs between the groups. Further research to understand the nature and possible mechanisms underlying this difference is warranted as it may have implications for intervention strategies. In light of previous findings that greater post-acute PCS increases the risk of chronic PCS elevation (Fordal et al., 2022), the present results have a number of clinical implications. Firstly, coping style is less relevant for outcome by 8 weeks post-injury than levels of psychological distress and sex, suggesting that it may be appropriate to consider earlier PCS intervention for females with higher psychological distress, irrespective of their coping style. Further, the results suggest that individuals with mTBI who believe that more of their generic symptoms are due to their injury, are at higher risk of elevated post-concussion symptoms 8 weeks after injury and may also warrant consideration for earlier intervention.
